# Maternal HPV Infection: Effects on Pregnancy Outcome

**DOI:** 10.3390/v13122455

**Published:** 2021-12-07

**Authors:** Carmen Elena Condrat, Lidia Filip, Mirela Gherghe, Dragos Cretoiu, Nicolae Suciu

**Affiliations:** 1 Department of Obstetrics and Gynecology, Polizu Clinical Hospital, Carol Davila University of Medicine and Pharmacy, 8 Eroii Sanitari Blvd., 050474 Bucharest, Romania; drcarmencondrat@gmail.com; 2 Fetal Medicine Excellence Research Center, Alessandrescu-Rusescu National Institute for Mother and Child Health, 020395 Bucharest, Romania; nsuciu54@yahoo.com; 3 Dermatology Department, Victor Babes Clinical Hospital of Infectious and Tropical Diseases, 030303 Bucharest, Romania; lidia.filipsd@gmail.com; 4 Department of Nuclear Medicine, Alexandru Trestioreanu Oncology Institute, 022328 Bucharest, Romania; 5 Department of Cell and Molecular Biology and Histology, Carol Davila University of Medicine and Pharmacy, 8 Eroii Sanitari Blvd., 050474 Bucharest, Romania; 6 Division of Obstetrics, Gynecology and Neonatology, Carol Davila University of Medicine and Pharmacy, 8 Eroii Sanitari Blvd., 050474 Bucharest, Romania; 7 Department of Obstetrics and Gynecology, Polizu Clinical Hospital, Alessandrescu-Rusescu National Institute for Mother and Child Health, 020395 Bucharest, Romania

**Keywords:** HPV, pregnancy, preterm birth, spontaneous abortion, immunization

## Abstract

The human papilloma virus (HPV) infection, caused by a ubiquitous virus typically transmitted through the direct contact of infected organs, either through the skin or mucosa, is the most common sexually transmitted infection, placing young women at a high risk of contracting it. Although the vast majority of cases spontaneously clear within 1–2 years, persistent HPV infection remains a serious concern, as it has repeatedly been linked to the development of multiple malignancies, including cervical, anogenital, and oropharyngeal cancers. Additionally, more recent data suggest a harmful effect of HPV infection on pregnancy. As the maternal hormonal environment and immune system undergo significant changes during pregnancy, the persistence of HPV is arguably favored. Various studies have reported an increased risk of adverse pregnancy outcomes among HPV-positive women, with the clinical impact encompassing a range of conditions, including preterm birth, miscarriage, pregnancy-induced hypertensive disorders (PIHD), intrauterine growth restriction (IUGR), low birth weight, the premature rupture of membranes (PROM), and fetal death. Therefore, understanding the mechanisms employed by HPV that negatively impact pregnancy and assessing potential approaches to counteract them would be of interest in the quest to optimize pregnancy outcomes and improve child survival and health.

## 1. Introduction

Human papilloma virus (HPV) encompasses a group of common viruses responsible for the most widespread sexually transmitted infection (STI), which is frequently asymptomatic and self-limiting [1] and also associated with the development of anogenital and oropharyngeal malignancies [2,3]. A family of small, non-enveloped, double-stranded DNA viruses, *Papillomaviridae* comprises over 200 HPV types, identified on the basis of genomic differences detected by DNA sequencing [4]. Phylogenetically, papillomaviruses are grouped into 53 genera, of which five are infectious to humans: alpha, beta, gamma, mu, and nu [5]. Based on their oncogenic potential for cervical cancer, HPV strains have traditionally been classified as low-risk (LR) and high-risk (HR) HPVs [6]. The various types of epithelial disease that HPVs cause are linked not only to their different strategies of transmission and propagation within the epithelium, but also to their diverse interactions with the immune system. The evolution of papillomaviruses has enabled them to adapt to specific epithelial niches, with different types being linked to different diseases. Thus, depending on tropism, HPV has further been divided into two main groups, namely cutaneous and mucosal HPVs [7]. While cutaneous HPVs belong to all five genera affecting humans, mucosal HPVs belong to the genus *Alphapapillomavirus* [5] (Table 1).

### 1.1. Cutaneous HPV

Cutaneous HPVs are inclined to infect the hair follicle stem cells, thus facilitating persistent infection [17]. Clinically, cutaneous HPVs typically lead to the development of benign tumors such as common warts (verruca vulgaris) and papillomas. Exposure to cutaneous HPVs is frequent, with studies estimating that around 90% of individuals would test positive for beta HPVs [18]. Furthermore, it has been observed that members of the same family may share some of beta and gamma HPV types [19,20]. However, serological tests show that about half of infected people develop antibodies [21], probably owing to the fact that affected keratinocytes constantly self-renew and the viral load is relatively low in immunocompetent patients [22,23]. While generally responsible for innocuous lesions, cutaneous HPVs in immunocompromised individuals such as organ transplant recipients and HIV-positive patients significantly increase their risk of developing cutaneous squamous cell carcinoma (cSCC) [24,25]. Along the same lines, there is increasing evidence suggesting that cutaneous HPV infection in healthy individuals chronically exposed to UV radiation enhances their risk of developing cSCC [26,27]. Additionally, the very rare autosomal recessive hereditary skin disorder epidermodysplasia verruciformis (EV) causes an increased susceptibility to cutaneous HPVs, associating cSCCs on sun-exposed areas [28]. While generally limited to the skin, individual studies have documented the presence of cutaneous HPV in mucosal epithelia, highlighting the dual tropism of some strains [10,11].

### 1.2. Mucosal HPV

Mucosal HPVs are typically contracted through coital or non-coital sexual contact, with non-sexual transmission still disputed [29]. The most recent data suggest that almost all sexually active individuals have been or will be infected with at least one type of genital HPV [30,31]. The International Agency on Research on Cancer (IARC) has categorized HPVs into HR and LR, with HR types being capable of promoting precancerous and cancerous lesions in affected individuals [32]. LR-HPVs, such as HPV types 6 and 11, are responsible for external anogenital warts (condyloma acuminatum) that may either resolve spontaneously or increase in size and number, especially in immunocompromised patients or during pregnancy [33]. While benign, enlarged genital warts in pregnant women can hinder vaginal delivery and lead to the occurrence of recurrent respiratory papillomatosis in the infant [34]. HR-HPVs are comprised of 13–14 types, of which HPVs 16 and 18 are the most common, being the causative agents of around 70% of precancerous and cancerous cervical lesions [35,36]. Other anatomical regions prone to developing precancerous squamous intraepithelial lesions (SILs)/intraepithelial neoplasia (IN) and squamous cell carcinoma due to HR-HPV infection are the oropharynx, vulva, vagina, penis, and anus [37]. While over 90% of cervical HPV infections spontaneously resolve in 12 to 24 months, the risk of the infection becoming persistent is increased by external factors such as alcohol and tobacco consumption [38,39]. Additionally, host genetic factors can also hinder the viral clearance, thus promoting the development of cervical cancer. For instance, human leukocyte antigen (HLA) genes, based on their capacity to bind to HPV proteins, can either promote carcinogenesis [40,41,42,43] or act as protectors [44]. Similarly, various polymorphic sites of the tumor necrosis factor (TNF) genes have been associated with an increased risk of cervical cancer [45,46].

## 2. How Does HPV Operate

**Figure 1:**
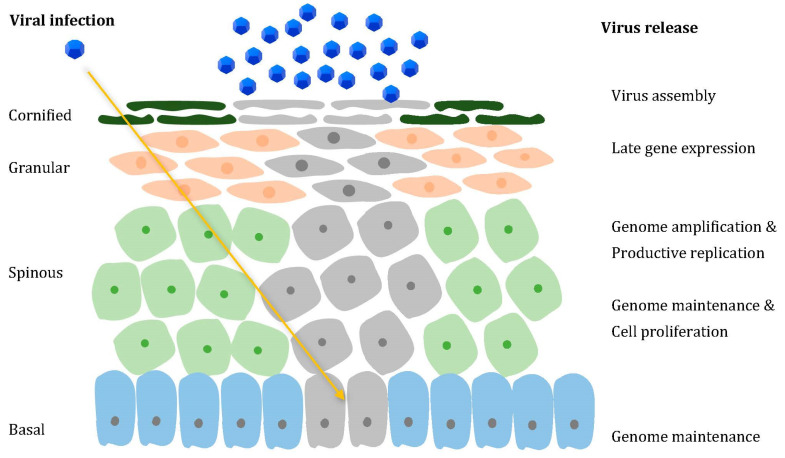
HPV life cycle. Through a microscopic wound, HPV reaches the basal layer of the stratified epithelium (yellow arrow), penetrating the cells within (affected cells are pictured in grey). The infected undifferentiated basal cells ensure viral DNA replication. The productive phase is gradually activated in the suprabasal layers, consisting of increased viral genome amplification, which is ensured by the ability of the E6 and E7 proteins to promote cell cycle re-entry. In the uppermost layers, away from immune surveillance, L1 and L2 expression facilitates encapsidation, thus allowing virion assembly and release.

HPV has a non-enveloped icosahedral structure with a diameter of approx. 50–60 nm, and its genome consists of circular double-stranded episomes of around 8000 base pairs, with 8 or 9 open reading frames (ORFs) [47]. The capsid is made up of two structural proteins, late 1 (L1) and late 2 (L2), necessary for virus assembly. The genome ORFs can be separated by functionality into three regions, as follows: the late (L) region encoding the L1 and L2 proteins, the early (E) region, encoding the proteins involved in viral replication, E1–E7, and the long control region (LCR), rich in cis-acting elements, which are essential for viral DNA replication and transcription [48]. The E1 and E2 viral proteins are necessary for viral replication, as, after binding to specific DNA sequences, they form a complex that launches progeny DNA synthesis [49], while E4 is thought to facilitate viral release and transmission [50]. Due to their role in cancer promotion, E5, E6, and E7 proteins are referred to as oncoproteins. They have been shown to successfully cooperate in tumorigenesis, not only by targeting negative cell cycle regulatory molecules, such as p53 and p105Rb tumor suppressors, but also by facilitating the process of them re-entering into the S-phase of differentiating cells [51]. As highly epitheliotropic viruses, it is currently believed that the viral life cycle starts at a wound site, following the infection of basal epithelial stem cells (Figure 1), which potentially contribute to lesion persistence [52]. As basal cells divide and gradually move away from the basement membrane to the suprabasal layers, normally they would undergo cell cycle withdrawal and progress towards terminal differentiation. However, infected cells are not able to stop cycling after division, in turn promoting persistent cell proliferation [53]. Afterwards, viral DNA amplification, along with capsid gene expression and virus assembly, successfully takes place in the suprabasal layers [54]. 

The expression of the HR-HPV E7 oncoprotein has been demonstrated to mediate not only the deterioration of p105, but also those of p107 and p130, thus promoting cell cycle entry and re-entry in the basal and suprabasal epithelial layers [55,56]. Additionally, E7 further promotes cell cycle entry and progression by inducing an extensive reprogramming of epigenetic marks [57]. Concurrently, the HR-HPV E6 oncoprotein leads to the degradation of p53, while also increasing telomerase activity and aiding telomere maintenance in order to support repeated cell divisions [58,59]. By supporting the re-entry into the S-phase within the suprabasal layers, the combined activity of E6 and E7 facilitates viral genome amplification. As genome amplification gradually shifts towards genome packaging in the upper dying keratinocytes, the production of E1, E4, and L1 increases, so as to allow cell cycle exit, viral encapsidation, and release.

## 3. HPV in Pregnant Women

The prevalence of HPV in pregnant women has been examined in several studies, with results collectively suggesting a higher risk of HPV infection among pregnant vs. non-pregnant women. In their systematic review gathering data from studies on HPV prevalence among pregnant patients, Liu et al. found an overall HPV prevalence of 16.82% in pregnant women vs. 12.25% in non-pregnant women [60]. More recently, Luo et al. performed a case–control study comprising pregnant women and age-matched non-pregnant women, and found a significantly higher HPV prevalence in the former group as opposed to the latter, 24.2% vs. 14.8%, respectively [61]. While most studies on pregnant women have searched for infection in the uterine cervix [60,61], HPV DNA has also been detected in the placenta [62,63,64,65], amniotic fluid [66,67], and umbilical cord [34], its presence in these tissues implying vertical transmission to the infant [68]. As plenty of requirements are needed in order to accommodate the products of conception, pregnancy is characterized by a myriad of adaptive changes, ranging from anatomical, cardiovascular, and metabolic, to hormonal and immunological [69]. To this extent, HPV infection and/or persistence have been speculated to be promoted by the modified immunological adaptations that are aimed at maintaining immune tolerance towards the semi-allogeneic fetus [70]. Additionally, HR-HPV glucocorticoid response elements (GREs) found in the viral upstream regulatory region can promote viral gene transcription and expression due to the increased steroid levels that typically define pregnancy [71,72].

Human trophoblasts have been shown to not only possess HPV receptors, but also facilitate HPV DNA replication [62,73]. Using trophoblast cell lines, You et al. further discovered that HPV infection leads to a decrease in trophoblast cell numbers and inhibits their ability to adhere to endometrial cells. By introducing the E7 oncogenic component, rapid cell death was visible, supporting the hypothesis that E7 promotes apoptosis in trophoblasts. Weakened endometrial cell binding could be observed by the introduction of both E6 and E7, with potential implications in embryo expulsion [74,75]. Later on, Boulenouar et al. evaluated the response of BeWo trophoblastic cell lines to being transfected with E5, E6, and E7 sequences and found decreased cell growth and adhesion, the latter presumably owing to their ability to inhibit E-cadherin, a molecule essential for cell–cell adhesion. In addition, trophoblast cells displayed accentuated migratory and invasive behaviors [76]. Further studies later highlighted that E5, in exerting its toxic effect, acts like a viroporin, meaning that it creates hydrophilic pores in cellular membranes, thus leading to apoptosis by osmotic stress [77,78].

The clearance of HPV during pregnancy has been shown to be decreased, with high viral loads additionally favoring persistence, which is in line with the hypothesis that pregnancy is defined by an altered immune response [79]. However, this trend can be observed in the first two trimesters, while the postpartum period is characterized by an increase in HPV clearance. This catch-up seems sufficient to compensate for the first two trimesters, as it has been observed that the cumulative incidence of HPV clearance is similar between pregnant and non-pregnant women during a 12-month period [80]. Studies have also shown that anti-HPV IgA antibodies are significantly increased in HPV-positive pregnant patients, both in the first trimester and early postpartum period. However, the local antibody production is rather weak, presumably owing to the partially suppressed local immune response [81]. 

## 4. HPV and Pregnancy Outcomes

Infectious pathogens during pregnancy have repeatedly been indicated to be responsible for adverse pregnancy outcomes, as well as a number of severe neonatal sequelae [82]. However, the involvement of HPV in the evolution and outcome of pregnancy is not quite clear, with studies reporting somewhat contradictory results: while some authors saw no relationship [83,84], others highlighted various adverse pregnancy outcomes, ranging from preterm birth [85], spontaneous abortion [86], the premature rupture of membranes [87], and pregnancy-induced hypertensive disorders [88] to intrauterine growth restriction [89], low birth weight [90], and fetal death [88]. A degree of contradiction between studies is, nevertheless, to be expected, if one takes into consideration the different sizes of the study samples, the more or less rigorous methodology, and the occasional lacking values for different variables (Table 2).

### 4.1. Preterm Birth

Preterm delivery refers to births occurring before 37 and after 20 weeks’ gestational age, and is typically linked to higher mortality rates, long-term morbidity, and hindered motor and cognitive development [102]. Worldwide figures show increasing preterm birth rates in the last two decades [103], one reason for this being the increase in multiple pregnancy rates following assisted reproductive technologies [104]. Other contributing factors include better chances of very premature babies surviving [105], a more advanced maternal age [106], and a rise in maternal obesity [107]. 

Both term and preterm labors occur as a result of the complex interaction between the mechanical uterine stretch and cell signaling pathways mediated by steroid hormones owing to the enhanced activity of the fetal hypothalamic–pituitary–adrenal (HPA) axis. Preterm delivery either occurs spontaneously, with intact or ruptured membranes, or is medically induced [108]. While the exact mechanism responsible for triggering preterm delivery cannot be established at all times, multiple factors are taken into consideration, including intrauterine infection/inflammation, uterine ischemia/hemorrhage, the overdistension of the uterus (e.g., multiple pregnancy, hydramnios), inadequate maternal immunological tolerance to the semi-allogeneic fetus, incompetent cervix, maternal stress, and hormonal disorders [109,110,111]. Furthermore, maternal/fetal conditions such as preeclampsia, gestational diabetes, fetal distress, or fetal anomalies are common reasons for indicated preterm delivery [112].

Intrauterine infection oftentimes gives rise to preterm birth by activating the innate immune system, which uses pattern recognition receptors (PRRs) to identify pathogen-associated molecular patterns (PAMPs) on microorganisms, and then induce the release of proinflammatory and chemotactic cytokines. Prostaglandin synthesis is consequently stimulated, which, in turn, promotes uterine contractility [110]. Infections may either ascend from the endocervical canal, reach the placenta by hematogenous dissemination through the maternal blood, or spread retrogradely from the abdominal cavity through the fallopian tubes [113]. While the link between preterm labor and bacteria such as *Ureaplasma urealyticum*, *Mycoplasma hominis*, *Fusobacterium* spp. and *Streptococcus agalactiae* has been studied and confirmed [114,115], evidence of the involvement of viral infections in preterm birth is limited, especially since viral infections leave few characteristic or specifically recognizable traces [63,116]. 

One potential mechanism for how HPV infection affects the outcome of pregnancy is through modifications in the vaginal microbiota that set off an immuno-inflammatory response initiating preterm labor [96]. Additionally, by infecting the trophoblast cells where it can easily replicate, HPV causes placental distress, thus contributing to preterm labor [93]. In their retrospective cohort study on 2153 women, Cotton-Caballero et al. have found that the rates of preterm delivery resulting from preterm the premature rupture of membranes (PPROM) increased [91]. Similarly, in their meta-analysis, Huang et al. have found that the risk of HPV-positive pregnant women delivering preterm was twice as great as the risk of those who were HPV negative [93]. Other authors reported comparable findings [62,94]. Furthermore, the more recent HERITAGE cohort study conducted by Niyibizi and colleagues looked at 899 pregnant women, and found that persistent HR-HPV infection led to a significant increase in the risk of preterm birth [95]. Likewise, the recently published retrospective population-based register study conducted by Wiik et al. highlighted the increased risk of preterm delivery among women with HPV infection and/or cervical intraepithelial neoplasia (CIN), especially if previously treated [96]. However, despite these hypotheses, not all studies have proven this association. Contributing to controversy in the field is Aldhous’ recent data-linkage study, which found that high-grade cervical lesions due to HPV increased the risk of preterm birth, but not HPV infection alone [85]. Moreover, in their prospective case–control study carried out on 271 patients, Ambühl et al. found no link between placental HPV, regardless of type, and spontaneous preterm labor [97]. Subramaniam et al. also could not find a link between maternal HPV infection and preterm delivery in their retrospective cohort study on 2321 patients [84]. Naturally, limiting factors are present in all studies, ranging from the relatively small size of the cohorts to the number of tests performed during pregnancy and sample collection. Therefore, in the pursuit of better reproductive outcomes, more scientific evidence regarding the involvement of HPV in preterm delivery should be gathered. 

### 4.2. Miscarriage

Pregnancy loss describes the death of the fetus and may refer to miscarriage (spontaneous abortion) when it occurs before 28 weeks’ gestational age/with a birth weight less than 1000 g/with a crown-to-heel length less than 35 cm, or stillbirth when it arises after this age, or when the fetus weighs over 1000 g or measures over 35 cm in length [117]. While the exact etiology cannot always be identified, it is estimated that over half of miscarriage cases are related to chromosomal anomalies [118]. Other causes include poor maternal health and extreme weights before pregnancy, uterine abnormalities, early exposure to teratogens, alcohol and/or tobacco, and infection during pregnancy [119,120]. The reduced number of natural killer cells resulting in a mild immunosuppression experienced during pregnancy is thought to stand at the root of pregnant women’s increased susceptibility to infections, including viral ones [121]. As pregnant women carry an increased risk of HPV infection [60], with HPV DNA being identified not only in the cervix [94,122], but also in the placenta [123,124], amniotic fluid [66], and fetal membranes [125], the question of spontaneous abortion has been posed repeatedly. However, the conclusions are controverted. For instance, Ambühl’s systematic literature search highlighted a higher HPV prevalence among women who had suffered a spontaneous abortion, without clearly attributing it a causative role. Specifically, while placental tissue samples from normal pregnancies were HPV positive in 8.3% of cases, pregnancies that had ended up in spontaneous abortion were HPV positive in 24.9% of cases [98]. These findings reflect previous discoveries, such as Hermonat’s work, who found higher rates of HPV-positive samples in spontaneously aborted products of conception in comparison with elective abortions [123]. Similarly, Bober et al. have observed higher HR-HPV rates in trophoblast cells from pregnancies ending in spontaneous abortion than normal pregnancies, highlighting the possibility of a hematogenous infection route [126]. Conversely, other studies have yielded the opposite results: Conde-Ferráez found no link between cervical HPV infection and the risk of spontaneous abortion [83]. Furthermore, looking at the relationship between recurrent miscarriage and HPV infection, Ticconi et al. found lower HPV rates among patients with recurrent miscarriage. They suggested that the increased immune reactivity responsible in part for the recurrent pregnancy loss is somehow protective against HPV infection [127]. Overall, studies examining the association between maternal HPV and spontaneous abortion report contradictory results, which, on top of this, are also limited by the relatively small sample sizes. As miscarriage remains the most common adverse pregnancy outcome [128], there is a clear need for further studies regarding this issue. 

### 4.3. Preeclampsia

Preeclampsia or pregnancy-induced hypertension (PIH) is a potentially severe complication that may arise either in the second part of pregnancy or postpartum period, consisting of the onset of high blood pressure and end organ damage, that may be associated with increased proteinuria. While both maternal and fetal outcomes are generally favorable in mild cases, morbidity and mortality risks remain elevated in the more severe cases. Additionally, preeclampsia patients carry the risk of later succumbing to cardiovascular and/or renal disease [129]. A systematic review analyzing worldwide preeclampsia rates has found that the condition arises in almost 5% of pregnancies [130]. Risk factors for preeclampsia include a past pregnancy complicated by preeclampsia [131], pre-existing hypertension [132], diabetes [133], autoimmune disorders, such as systemic lupus erythematosus and antiphospholipid syndrome [131], and chronic kidney disease [134]. However, it is becoming increasingly apparent that the inflammation and endothelial dysfunction resulting from infection might play a role in preeclampsia [135,136]. For instance, the link between preeclampsia and periodontitis has been widely reported [137,138], as well as acute infections such as urinary tract infections [139]. Chronic maternal infections, such as cytomegalovirus [140], *Chlamydia pneumoniae* [141], and *Helicobacter pylori* [142,143], have also been demonstrated to play a role in preeclampsia.

Several studies have examined the potential involvement of HPV infection in preeclampsia, with opposing results. In 2008, Gomez et al. performed a case–control study of 108 subjects and found that HPV DNA prevalence in placental samples from preeclampsia cases was similar to the one in the control group [62]. Later on, Cho et al. carried out a cross-sectional study on 311 pregnant women and found that HR-HPV (identified at six weeks postpartum) had no influence over the risk of a pregnant woman developing preeclampsia [87]. Likewise, in their retrospective cohort study on 15,357 women, Nimrodi et al. reported similar findings. However, their study included Pap smears obtained up to 2 years before delivery or in the first trimester of pregnancy [99]. On the other hand, McDonnold et al. performed a similar retrospective cohort study on a smaller sample size, comprising 942 cases, where they obtained Pap smears at entry to prenatal care. They found that HR-HPV appeared to contribute about a twofold increase in preeclampsia risk [100]. Slatter et al. later had similar findings, by examining placental HR-HPV [65]. Overall, little research has been conducted on this matter, meaning that HPV infection cannot yet be established as a preeclampsia risk factor.

### 4.4. Intrauterine Growth Restriction

Intrauterine growth restriction (IUGR) occurs when the fetus does not grow as expected according to its gestational age. IUGR encompasses fetal growth restriction (FGR) and small for gestational age (SGA) [144]. While SGA fetuses are constitutionally small, FGR is diagnosed when a fetus’ weight lies below the 10th percentile for its gestational age (GA) as a result of a pathological factor, and it is a very important cause of perinatal mortality and morbidity [144,145]. Maternal and placental factors, along with genetics, contribute to fetal growth. While constitutionally small babies are normally developed and adequately proportioned, babies with FGR are typically undernourished and/or dysmorphic [146]. A combination of maternal, fetal, and placental factors commonly leads to FGR. Maternal causes include medical conditions such as chronic hypertension, diabetes, cardiovascular disease, autoimmune disorders, and infections, along with smoking, drinking alcohol, and having a low preconception body mass index [147]. Placental dysfunction is another leading cause of FGR and occurs as a result of preeclampsia, abnormal blood vessels, or thrombophilia, or it can be idiopathic [148]. Fetal causes broadly include genetic abnormalities, birth defects, metabolic disorders, and in utero infections [149]. 

Conclusively proved maternal infections that lead to FGR include the TORCH group, consisting of *Toxoplasma gondii*, rubella, cytomegalovirus, herpes simplex virus types 1 and 2, and other agents. Evidence regarding the negative impact on pregnancy, thus resulting in IUGR, has also been accumulating for varicella-zoster virus [150], *Treponema pallidum* [151], *Plasmodium falciparum* [152], and parvovirus B19 [153]. The most important mechanism in infectious IUGR is thought to be the fetal inflammatory response syndrome brought about by the uteroplacental infection and inflammation [115]. Additionally, some infectious agents are able to induce the cytolysis of target cells, thus damaging different fetal organs [149]. More recent research has further recognized the role of maternal HPV infection in IUGR. For instance, in their study analyzing 54 pregnant women in the third trimester, Karowicz-Bilińska et al. have shown a clear association between maternal HR-HPV infection certified by positive Pap smears and viral DNA from placental tissue and IUGR [154]. Further on, Slatter et al. investigated a cohort of 339 pregnancies by looking at decidual and endometrial HPV and found that HPV infection was correlated not only with higher rates of FGR, but also prematurity and acute chorionamnionitis [65]. On a similar note, in 2011, Ford et al. looked at preconception risk factors for lowered birth weight. Their prospective study on 585 women highlighted that, along with low levels of vitamin D and omega 3 fatty acids, positive Pap smears seemed to increase the risk of having a baby weighing less than the third percentile [155]. Following this small study, Ford et al. later investigated this association in their data-linkage study, comprising 31,827 women. They found that mothers with abnormal Pap smears were at an increased risk of giving birth to babies beneath the third percentile, with very low birth weight (VLBW), independent of other risk factors [89]. 

### 4.5. Premature Rupture of Membranes

Premature rupture of membranes (PROM) defines the breaking open of the amniotic sac before labor. Should this happen before 37 weeks’ GA, it is referred to as the preterm premature rupture of membranes (PPROM) [156]. Term prelabor rupture of membranes (TPROM) is a complication that arises in around 10% of pregnancies, entailing both maternal and neonatal risks [157]. Complications that might arise as a result of PROM include placental abruption, pulmonary hypoplasia in the fetus, umbilical cord prolapse and/or compression, leading to fetal distress due to the lack of oxygen [158]. However, infection remains the most severe consequence of TPROM and PPROM, for both mother and baby, with reports stating that chorioamnionitis may occur in almost one-third of PROM-affected pregnancies [159]. On the other hand, infection can also be the cause of PROM, with uterine, cervical, and vaginal infections being among the most important risk factors. Other factors linked to PROM include uterine distension such as in the case of polyhydramnios and multifetal pregnancies, cerclage, tobacco use, and vaginal bleeding [160]. 

Concerning the link between HPV and PROM, several studies have analyzed its involvement in this condition. For instance, Cho et al. have specifically looked at the incidence rates of PROM in both HR-HPV-positive and HR-HPV-negative women. Their study found that 27.3% of HR-HPV-positive women experienced PROM, compared with 14.2% in the HR-HPV-negative group (*p* = 0.029), thus highlighting the increased risk of PROM that HPV entails [87]. Somewhat similarly, both Cotton-Caballero’s and Pandey’s studies revealed an increased risk for PPROM in HPV-positive pregnant women, but not TPROM [91,161]. In a similar manner, Wiik’s retrospective study on 400,583 singleton deliveries from previously nulliparous women found that HPV infection (be it by cervical HPV testing or cytology) was significantly associated with PPROM and TPROM [96]. On the other hand, in their case series comprising 20 patients, despite the small sample, Giambanco et al. found that HPV did not increase the risk of PPROM [101].

### 4.6. Fetal Death

The spontaneous intrauterine death of the conceptus at any stage of pregnancy defines fetal death. While the etiology remains unknown in around a quarter of cases, one prospective study revealed that almost two-thirds of cases can be attributed to placental dysfunction [162]. Other causes broadly include obstetric complications, infections, congenital anomalies, hypertensive disorders, poorly managed diabetes, and the use of tobacco, marijuana, or stimulants such as cocaine and amphetamines [163]. Slatter et al. have been the first to observe the association between maternal HPV infection and fetal demise, although the link has not been thoroughly examined. More precisely, 81% (13/16) of fetal deaths were from HPV-positive mothers with no prior medical conditions, enabling the authors to establish a relationship between viral infection and fetal demise [65]. Shortly afterwards, a further study carried out on a larger sample size by Subramaniam et al. had similar findings. However, as fetal death in the latter study was a tertiary outcome, the authors could not draw a firm conclusion regarding this association [84].

## 5. Immunization of Pregnant Women

Vaccines are largely acknowledged as one of the greatest public health triumphs, due to their efficiency in reducing the spread of, and even eradicating, numerous infectious illnesses [164]. Still, while childhood immunization rates are generally high, parents and caregivers continue to voice concerns regarding vaccine safety [165]. As a result, adult vaccination rates fall behind those of children, and only approximately a third of pregnant women receive vaccinations as recommended throughout their pregnancy [166]. However, ensuring vaccine safety remains an important matter for public health, especially in light of the changing vaccination landscape [167]. Cervical cancer and other HPV-related illnesses pose important worldwide public health issues, prompting the WHO to reiterate the recommendation that HPV vaccinations be included in national immunization programs [168]. Since it accounts for 84% of all HPV-related malignancies, cervical cancer prevention should remain the most important reason behind HPV vaccination, and immunizing females before the start of their sexual life is one of the most effective means of prevention [169,170]. Additionally, after carrying out a thorough population-level analysis, Yuill et al. have recently found the first evidence of a reduction in negative pregnancy outcomes, such as preterm birth and low birth weight, in women previously exposed to the HPV vaccination [171]. 

To date, the HPV vaccination scheme approved by the Food and Drug Administration includes three recombinant vaccines: bivalent (Cervarix–types 16 and 18), quadrivalent (Gardasil–types 6, 11, 16, 18), and nonavalent (Gardasil-9 types 6, 11, 16, 18, 31, 33, 45, 52, and 58) [172,173,174]. Using recombinant DNA technology, the capsid proteins of the HPV boosted by an adjuvant are assembled into virus-like particles (VLPs) capable of triggering a more enhanced immune response than a natural infection [175,176]. In order to benefit from the full coverage of the vaccine, two or three doses should be administered, depending on the age of the patient (0, ± 2 months, and at 6 months, especially when the vaccination scheme starts after the age of 15, or for immunocompromised patients) [177,178]. Aiming to achieve high levels of anti-HPV antibodies, thus ensuring long-term immunity, the quadrivalent vaccine is administered in most high-income countries as part of a routine immunization schedule [179,180]. Moreover, current recommendations regarding HPV immunization support the vaccination of women who have already tested positive for HPV, since it will help protect against certain HPV types that they have not been exposed to [181]. Additionally, Valasoulis et al. have recently demonstrated that, by vaccinating women with low-grade cytological anomalies against HPV-16, -18, -31, and -33, they benefited from the earlier clearance of HPV [182]. This is especially relevant for women planning to get pregnant, as they would benefit most from potentially avoiding cervical conization, due to the the increased risk of subsequent preterm labor that it likely entails [183]. 

Since the vaccine may be delivered throughout a woman’s reproductive life for a variety of reasons, there is a danger of unintentional immunization either during or just before pregnancy in many situations [184]. Thus, the subject of HPV vaccination and pregnancy is a hot topic in the world of immunology, virology, and the everyday standard practice of gynecologists, due to concerns related to safety and possible harmful side effects [185]. Given the fact that the rate of unplanned pregnancies worldwide ranges between 18 and 47% depending on geopolitical status, further research is needed to convey the full spectrum of implications of HPV vaccination in pregnant women [186,187,188]. However, due to a lack of feasibility in terms of ethics, administering the vaccines to pregnant women has not yet been approved. HPV vaccination is currently not recommended during pregnancy, according to the WHO, the Canadian Advisory Committee on Immunization, and vaccine producers [168]. Nevertheless, research carried out by the manufacturers of HPV vaccines for regulatory purposes revealed that most adverse reactions occurring in HPV-vaccinated pregnant women were comparable to those in the general population. As such, in terms of maternal safety, The European Medicines Agency investigated postural orthostatic tachycardia syndrome and complex regional pain syndrome and found no evidence of a causal relationship between vaccinations and these side effects [189]. Moreover, several studies on this matter concluded that pregnant women who received the HPV vaccine did not experience the onset of acute or chronic diseases or major adverse events more than women who did not receive the vaccine [190,191,192]. Regarding fetal outcomes, most of the data were focused on spontaneous miscarriage, with results suggesting a negligible correlation between vaccination and the risk of miscarriage [193,194,195]. Only one study discovered a mildly increased risk of miscarriage (14.7% vs. 9.2%, *p* = 0.031), yet it did not take into account variables such as parity, race, or socioeconomic status, which could have greatly influenced the results [196]. Ectopic pregnancies, congenital anomalies, or fetal death did not appear to occur more frequently in women receiving the HPV vaccine during pregnancy or within one month before the first day of the last menstrual period, compared to the general population [195]. Overall, the current scientific views of HPV vaccination during or prior to pregnancy indicate little or no evidence linking HPV vaccination and adverse outcomes of pregnancy; still, it is important to monitor and follow-up inadvertent administrations. Additionally, the remaining vaccine doses are still recommended to be postponed until after childbirth, since further studies are necessary to conclude the safety profile of pregnancy vaccination [197].

## 6. Conclusions

As highlighted throughout this review, HPV infection during pregnancy might negatively impact both maternal and infant health, increasing the risk of severe pregnancy complications, such as spontaneous abortion, preterm birth, preeclampsia, intrauterine growth restriction, premature rupture of membranes, and even fetal death. While absolute conclusions are impeded by potential bias and the relatively small number of studies, it is our firm belief that further research fully addressing this matter would confirm the existence of a causal relationship between HPV and adverse pregnancy outcomes. Moreover, the widespread implementation of HPV immunization programs should be initiated and continued, since it is expected to reduce not only cervical cancer rates, but also the risk of cervical conization-related preterm birth, as well as negative pregnancy outcomes related to HPV infection. 

## Figures and Tables

**Table 1:** HPV genera and properties.

Genus	Biological and Clinical Aspects
*Alpha-* *papillomavirus*	Mucosal and cutaneous lesions Comprised of 14 species (α1–α14) that include 65 HPV types [8] Molecular genotyping of HPV L1 gene in low-risk and high-risk groups
*Beta-* *papillomavirus*	Cutaneous lesions Comprised of 5 species (β1–β5) that include 54 HPV types [9] Reports of beta HPV in mucosal epithelia [10,11] Etiological role in non-melanoma skin cancer [12] Promotes the development of cSCC in EV patients [13]
*Gamma-* *papillomavirus*	Cutaneous lesions Comprised of 27 species (γ1–γ5) that include 98 HPV types [9] Reports of gamma HPV in mucosal epithelia [14,15]
*Mu-* *papillomavirus*	Cutaneous lesions Includes 3 HPV types [16]
*Nu-* *papillomavirus*	Cutaneous lesions Includes 1 HPV type [16]

L1 = major capsid protein; cSCC = cutaneous squamous cell carcinoma; EV = epidermodysplasia verruciformis.

**Table 2:** Studies addressing the impact of HPV infection on pregnancy outcome.

**Authors, Year**	**Study Type**	**Sample**	**HPV Detection**	**HPV Type**	**HPV History (e.g., Previous History of CIN, Genital Warts)**	**Conclusions**
Cotton-Caballero et al., 2017 [91,92]	Retrospective cohort study (2153 pregnant women)	Cervical samples	Cervical cytology HPV genotyping	HR-HPV (types 16, 18, 31, 33, 35, 39, 45, 51, 52, 56, 58, 59, and 68)	Patients with treated cervical dysplasia (conization, loop electrosurgical excision procedure, and cryotherapy) were included and adjusted for	HR-HPV infection led to an increase in PPROM and preterm birth resulting from PPROM, but not preterm birth without PPROM
Huang et al., 2014 [93]	Systematic review (8 studies)	Cervical samples	Cervical cytology HPV DNA testing (ISH, PCR)	HR-HPV LR-HPV undefined	Two studies in the meta-analysis included adjustment for prior cervical procedures	HR-HPV-infected women had an overall 2.55-fold increased risk of delivering prematurely
Zuo et al., 2011 [94]	Retrospective study (2480 cases)	Cervical samples Placental tissue	Cervical cytology Reflex HPV DNA testing via RNA-DNA hybrids Pathologic examination of the placenta	HR-HPV	Not specified	HR-HPV-related changes in cervical cytology were associated with preterm birth and placental abnormalities
Gomez et al., 2008 [62]	Case–control study (108 cases)	Placental tissue	HPV DNA testing (PCR) followed by HPV type confirmation via DNA sequencing	HR-HPV (types 16, 18) LR-HPV (types 6, 11)	Not specified	HR-HPV infection was correlated with placental abnormalities and preterm delivery HR-HPV infection did not increase the risk of preeclampsia
Niyibizi et al., 2021 [95]	Prospective cohort study (899 pregnant women)	Vaginal secretion samples Placental tissue	HPV DNA testing and genotyping (PCR)	HR-HPV LR-HPV	7.1% of women had previously undergone treatment for CIN	Persistent vaginal HPV-16/18 infection and placental HPV infection were associated with an increased risk of preterm delivery Treatment for HR-HPV-related cervical dysplasia also increased the risk of delivering prematurely
Wiik et al., 2021 [96]	Retrospective population-based register study (400,583 pregnant women)	Cervical samples	HPV DNA testing Cervical cytology Cervical histology	Not specified	Women previously treated for CIN were excluded from this study	HPV infection identified via DNA testing was associated with a higher risk of PPROM than HPV infection, certified through cytology, without DNA testing Both positive cytology and positive HPV DNA testing were associated with an increased risk of preterm delivery, PROM, PPROM, and neonatal mortality
Aldhous et al., 2019 [85]	Data-linkage study (5598 pregnant women)	Cervical samples	HPV DNA testing Cervical cytology Cervical histology	HR-HPV LR-HPV undefined	No data regarding previous treatments for HPV-associated cervical disease	High-grade HPV-related cervical disease was associated with an increased risk of preterm birt hLow-grade HPV-related cervical disease and HR-HPV infection with no disease did not increase the risk of preterm delivery
Ambühl et al., 2017 [97]	Prospective case–control study (271 pregnant women)	Placental tissue	HPV DNA detection via nested PCR, followed by HPV genotyping via CISH	HR-HPV LR-HPV	Patients with genital warts, cervical dysplasia/carcinoma in situ/cancer were included in this study	Placental HPV infection was more frequent among women with history of cervical cancer The prevalence of placental HPV was similar in both complicated and uncomplicated pregnancies
Subramaniam et al., 2016 [84]	Retrospective cohort study (2321 pregnant women)	Cervical samples	HPV DNA testing Cervical cytology	HR-HPV	Women previously treated for CIN were excluded from this study	HR-HPV infection did not increase the risk of developing pregnancy-induced hypertensive disorders (PIHDs) and/or delivering prematurely HR-HPV infection was associated with an increased risk of placental abruption and severe preeclampsia
Ambühl et al., 2016 [98]	Systematic literature search (42 studies)	Cervical samples Placental tissue	HPV DNA testing (PCR, DNA chip, hybrid capture, Southern blot) Pathologic examination of the placenta	HR-HPV LR-HPV	Studies either included or excluded women with HPV-related lesions One-third of studies did not specify this aspect	Overall, the authors concluded that HPV infection could increase the risk of spontaneous abortion or spontaneous preterm delivery
Conde-Ferráez et al., 2013 [83]	Case–control study (127 cases)	Cervical samples	HPV DNA testing (PCR) HPV genotyping (NMPCR)	HR-HPV LR-HPV	Not specified	No significant association between HPV infection and spontaneous abortion was found
Cho et al., [87]	Cross-sectional study (311 cases)	Cervical samples	HPV DNA testing (via RNA–DNA hybrids)	HR-HPV	Not specified	HR-HPV infection was associated with an increased risk of PROM at term HR-HPV infection was not linked to a higher risk of preeclampsia
Nimrodi et al., 2018 [99]	Retrospective cohort study (15,357 cases)	Cervical samples	Cervical cytology	Not specified	Not specified	HPV infection did not increase the risk of developing preeclampsia, cervical insufficiency, placental abruption, PROM, PPROM, or preterm delivery
McDonnold et al., 2013 [100]	Retrospective cohort study (942 cases)	Cervical samples	Cervical cytology HPV DNA testing	HR-HPV	Not specified	HR-HPV appeared to contribute an approximately two-fold increase in preeclampsia risk
Slatter et al., 2015 [65]	Cross-sectional study (339 cases)	Placental tissue	Pathologic examination of the placenta	HPV DNA testing (IHC, CISH, PCR)	History of cervical HPV infection was available for two=thirds of women	Placental HPV infection was linked to negative pregnancy outcomes and complications, such as preterm birth, fetal growth restriction, fetal demise, diabetes, and preeclampsia Previous cervical HPV infection was a risk factor for developing placental HPV infection
Ford et al., 2019 [89]	Data-linkage study (31,827 pregnant women)	Cervical samples	Cervical cytology	Not specified	Women with previous abnormal cervical cytology were included in the study and adjusted for	Abnormal Pap results were an independent risk factor for IUGR, and especially very low birthweight
Giambanco et al., 2020 [101]	Case series (20 cases)	Cervical samples	Cervical cytology HPV DNA testing (multiplex RT-PCR)	HR-HPV LR-HPV (but not specified)	Women with previous history of CIN and/or abnormal Pap smears were excluded from the study	HPV infection was not associated with adverse pregnancy outcomes such as miscarriage, PPROM, and preterm birth

CIN—cervical intra-epithelial neoplasia; PCR—polymerase chain reaction; RT-PCR—real-time polymerase chain reaction; NMPCR—nested multiplex polymerase chain reaction; ISH—in situ hybridization; CISH—chromogenic in situ hybridization; IHC—immunohistochemistry.

## Data Availability

No new data were created or analyzed in this study. Data sharing is not applicable to this article.
